# Prevalence of Community-Acquired Pyoderma in Dermatological Outpatient Department of a Tertiary Care Hospital

**DOI:** 10.31729/jnma.4430

**Published:** 2019-06-30

**Authors:** Rupak Bishwokarma Ghimire, Khilasha Pokharel, Sanjubabu Shrestha

**Affiliations:** 1 Department of Dermatology, Kathmandu Medical College, Sinamangal, Kathmandu, Nepal; 2 Department of Microbiology, Kathmandu Medical College, Sinamangal, Kathmandu, Nepal

**Keywords:** *drug resistance*, *pyoderma*, *skin infections*, *staphylococcus*

## Abstract

**Introduction:**

Pyoderma is defined as any purulent skin disease and represents infections in epidermis and dermis or hair follicles. This study aims to find out the prevalence of community-acquired pyoderma in dermatological outpatient department of a tertiary care hospital.

**Methods:**

A descriptive cross-sectional study was carried out among patients who presented at dermatology outpatient department of Kathmandu Medical College Teaching Hospital between December 2018 and March 2019 after ethical clearance from institutional review committee. Convenience sampling method was done. Data was collected and analysis was done using SPSS software, point estimate at 95% CI was calculated along with frequency and proportion for binary data.

**Results:**

Out of 385 cases, 72 (18%) cases were of community-acquired pyoderma. Prevalence of community-acquired pyoderma is 72 (18%). Primary pyoderma was seen in 49 (12.72%) mainly folliculitis 17 (4.41%), furunculosis 16 (4.15%), impetigo 6 (1.55%), abscess 6 (1.55%) and bacterial paronychia 4 (1.03%). Staphylococcus aureus was the most common organism isolated in 42 (58.3%) cases and Staphylococcus epidermidis was isolated in 3 (4.17%) cases. Staphylococcus aureus was most sensitive to Vancomycin 42 (100%) followed by Gentamycin 40 (95.2%), Ciprofloxacin 40 (95.2%) and Ceftriaxone 40 (95.2%). Highest resistance was seen to Azithromycin in 13 (30.95%), followed by Cloxacilllin in 11 (26.19%). Males were affected predominantly in 49 (68.06%) as compared to females in 23 (31.94%).

**Conclusions:**

Prevalence of community-acquired pyoderma is high among patients visiting dermatological outpatient departments of a tertiary care hospital compared to other studies. Antibiotic resistance of commonly used antibiotics are increasing and thus proper culture and sensitivity reports may be required to guide our treatment.

## INTRODUCTION

Pyoderma is defined as any purulent skin disease and represents bacterial infections in epidermis, dermis or in hair follicles.^1^ Primary pyodermas are impetigo, follicultis, furuncle, carbuncle, ecthyma, erthyrasma, and sycosis barbae. Secondary pyodermas constitute various other dermatoses infected with organisms.^2^ Pyoderma is one of the commonest clinical conditions encountered in dermatological practice.^3^ Staphylococcus aureus and Streptococcus pyogenes are the common causative agents.^4^ Many factors influence the prevalence of pyodermas- poverty, malnutrition, overcrowding, poor hygiene and climatic conditions leading to its higher incidence in people of lower socioeconomic status from developing countries.^5^ Antibiotic sensitivity patterns differ from region to region and with progress of time. The increasing resistance to the antibiotics is a challenge to treating doctors.^6^ Detailed knowledge about the causative organisms and antibiotic susceptibility pattern should be known for successful treatment.^1^

This study aims to find out the prevalence of community-acquired pyoderma in dermatological outpatient department of a tertiary care hospital along with the clinical profile, bacteriological study and antibiotic sensitivity.

## METHODS

This descriptive cross-sectional study was carried out in patients visiting dermatology outpatient department of Kathmandu Medical College Teaching Hospital from December 2018 to March 2019. Ethical clearance was taken from Institutional Review Committee (IRC) Ref no. 2311201815.


$$\text{n}={\text{Z}}^{2}\times (\text{p}\times \text{q})/{\text{d}}^{2}$$

where,

n = sample size
p = prevalence, 50%
q = 1-p
d = margin of error, 5%
Z = 1.96 at 95% CI

Hence, calculated sample size was 385. Convenient sampling was used for the study.

Pyoderma was diagnosed by presence of pus on examination and for crusted lesions, removal of crusts to see active pus collection and pus was collected by sterile techniques for gram strain morphology, culture and antibiotic sensitivities. All patients diagnosed with pyoderma of the skin with all ages & gender, diagnosis verified by two dermatologists, were included in our study. Patients with history of hospital admission in last 48 hours history of hemodialysis, surgery, residence in a long-term care facility or hospitalization during the previous 12 months and indwelling catheter or percutaneous device in last 48 hours excluded from our study.

Potential bias could have been due to presence of normal skin flora are similar to the pathogens. To reduce this, the affected skin lesions were swabbed with alcohol and the pus collected by using a sterile cotton swab. They were then processed as per the standard protocol for the isolation of aerobic bacteria in coordination with a consultant from department of microbiology.

Staphylococcus aureus was identified based on Gram's stain morphology, colony characteristics and positive catalase and coagulase tests. Antibiotic sensitivity test was performed, by using Kirby Bauer Disc Diffusion. The antimicrobials tested were Azithromycin, Gentamicin, Ciprofloxacin, Cloxacillin, Vancomycin, Ceftriaxone and Cefixime. Staphylococcus aureus ATCC 25923 used as a control. Methicillin resistance was detected by using 1 mg oxacillin discs.

Selection bias and information bias has been minimized as possible. Data analysis was done using SPSS software package (SPSS 13.0 Inc., Chicago, IL, USA), point estimate at 95% CI was calculated along with frequency and proportion for binary data.

## RESULTS

Out of 385 cases seen by two dermatologists during the study period, 72 (18%) cases were diagnosed with community-acquired pyoderma. Prevalence of community-acquired pyoderma is 72 (18%) at 95% CIO. Different spectrum of diseases in pyoderma were seen- primary pyodermas 49 (12.72%), mainly folliculitis 17 (4.41%), furunculosis 16 (4.15%), impetigo 6 (1.55%), abscess 6 (1.55%) and bacterial paronychia 4 (1.93%).

Among the secondary pyodermas seen in 23 (5.97%) cases, most common case was after insect bite reaction in 6 (1.55%), followed by infected eczema in 4 (1.03%), miliria in 4 (1.03%), scabies 2 (0.52%), keloid 2 (0.52%) and chicken pox 1 (0.25%) (Table 1).

**Table 1.** Clinical pattern of pyoderma.

Primary Pyodermas	n (%)	Secondary Pyodermas	n (%)
Folliculitis	17 (4.41%)	Insect bite	6 (1.55%)
Furunculosis	16 (4.15%)	Infected Eczema	4 (1.03%)
Impetigo	6 (1.55%)	Miliaria	4 (1.03%)
Abscess	6 (1.55%)	Scabies	2 (0.52%)
Bacterial Paronychia	4 (1.03%)	Keloid	2 (0.52%)
		Chicken Pox	1 (0.25%)

Among the cases diagnosed as pyoderma, maximum number of cases were in age group below 10 years: 22 (30.56%) cases, followed by ages between 2130 years:16 (22.22%), 11-20 years: 14 (19.44%), 41-50 years: 3 (4.17%), 61-70 years: 3 (4.17%), 71-80 years: 2(2.78%) and 51-60 years: 1(1.38%). Males were affected predominantly in 49 (68.06%) as compared to females in 23 (31.94%).

**Figure 1.**
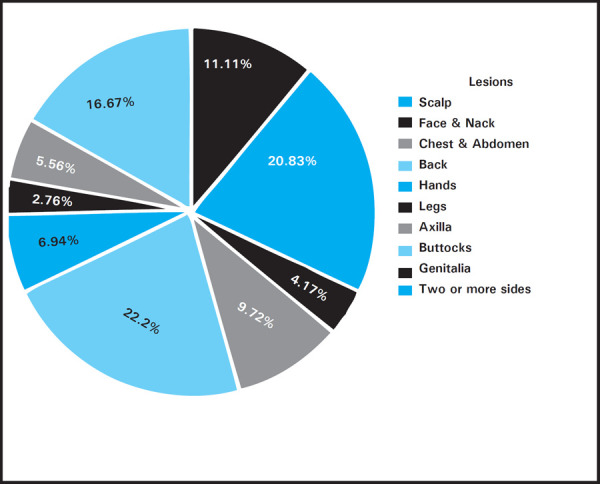
Body part affected by the skin lesions.

Duration of the lesions at presentation to the hospital were between 3-7 days in 32 (44.44%), more than 7 days in 23 (31.94%) and less than 3 days in 17 (23.61%). The most common body part involved were legs 22.22% (n=16), face & neck 20.83% (n=15), two or more sites involved in 16.67% (n=12) depicted (Figure 1).

Bathing habits were also asked to the patients, where only 16 (22.22%) said they bathe daily, 12 (16.67%) alternate days bathing, 24 (34.72%) twice a week, 18 (25%) patients said they bathe once a week and two patients said they bathe in interval of more than a week.

**Figure 2.**
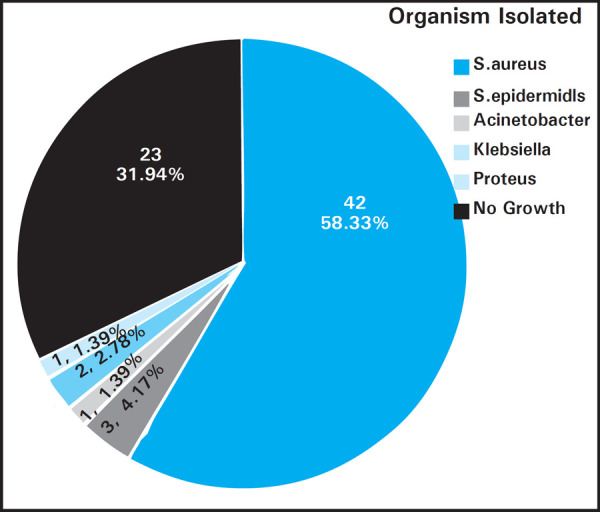
Organisms isolated.

Staphylococcus aureus was the most common organism isolated in 42 (58.3%). Staphylococcus epidermidis was isolated in 3 (4.17%) cases, Klebsiella in 2 (2.6%) cases, Acinetobacter and Proteus in 1 (1.39%) cases. No growth was seen in 23 (31.9%) cases (Figure 2).

Staphylococcus aureus was most sensitive to Vancomycin 42 (100%) followed by Gentamycin 40 (95.2%), Ciproflixacin 40 (95.2%) and Ceftriaxone 40 (95.2%). Highest resistance was seen to Azitromycin in 13 (30.95%), followed by Cloxacilllin in 11 (26.19%) (Table 1). Both samples of Staphylococcus epidermidis were resistant to Cloxacillin, while one each was resistant to Ceftriaxone and Azithromycin respectively. Acinetobacter was resistant to Azithromycin & Cefixime but sensitive to other antibiotics. Proteus mirabilis isolated was resistant to Cefixime and sensitive to other antibiotics (Table 2).

**Table 2.** Antibiogram of Staphylococcus aureus.

S. No	Antibiotic	Sensitive Number (%)	n (%)
1	Cloxacillin	31 (73.8)	11 (26.19)
2	Azithromycin	29 (69)	13 (30.95)
3	Gentamicin	40 (95.2)	2 (4.76)
4	Ciprofloxacin	40 (95.2)	2 (4.76)
5	Ceftriaxone	40 (95.2)	2 (4.76)
6	Cefixime	32 (76.1)	10 (23.8)
7	Vancomycin	42 (100)	0

## DISCUSSION

Several studies have stated that bacterial skin infections may account for up to 17% of clinical visits.^7^ This statement coincides with findings from our study, where the prevalence of pyoderma among dermatological visits were found to be 18%. In different studies the prevalence has ranged from 2.55% to 29.8%.^8,9^ In a community-based study among children, prevalence of pyoderma was 4.5%.^10^ A slight higher percentage in our study was probably because the study was done among patients who came to seek for healthcare, rather than a community study.

The findings from our study showed higher prevalence in younger age groups less than 10 years 22 (30.56%). Out of 200 cases of pyoderma in Gwalior, India, maximum patients were in the age group of less than 10 years 48%, followed by the age group of 21-30 years 21%, 11-20 years 17%, 31-40 years 9%, 5160 years 2.5%, 41-50 years 1.5%, and >60 years 0.5%. The prevalence rate was higher in male 61.2% of patients compared with females 38.5% of patients.^11^ These findings coincided with our observation where males were affected predominantly in males 68.06 % and in females 31.94%.

In a study carried out in 61 patients in a tertiary center in India, primary pyoderma accounted for 19.67% cases while 80.33% cases were of secondary pyoderma in contrary to our study where primary pyodermas were more common in 68.05% cases. Folliculitis accounted for 23.61 % and the most common disease in our study followed by furunculosis 23.61 %, impetigo 4.32%, abscess 4.32% and bacterial paronychia in 5.55% whereas, impetigo was the commonest entity seen 14.75% of cases in this study. Among the secondary pyodermas, secondarily infected pemphigus vulgaris was the commonest, being seen in 39.34% whereas insect bite reaction in 4.32% was commonest cause of secondary pyoderma in our study. In the same study, Staphylococcus was the most common organisms to be isolated in 59.01 % cases. This finding was similar to findings of our study where Staphylococcus was seen in 58.3% cases. The other organisms isolated in this study were Klebsiella in 4.92% of cases, Streptococcus, Enterococcus and Proteus in 3.27% of cases each and Citrobacter and E.coli in 1.64% of cases each, more than one type of organism from 4.92% and no organism from 14.75% of cases.^12^ No growth was seen according to reports in 31.94% of cases.

In a similar study carried out in 2009, on 75 patients at Tribhuwan University Teaching Hospial, Nepal out of which 34.7% were females and 65.3% were males, different from our study with male preponderance. It was more common in age group 15-25 years with 33.3%, followed by 26-35 years in 22.7%. Lower limbs were commonest site to be affected in 29.3% which is similar to our finding. Staphylococcus aureus was the commonest organism to be isolated in 86.7% more than our findings. Ampicillin resistance was found to in 78.2% and Cloxacillin resistance was 5.8%.^13^ There seems to be a change in resistance pattern in last 10 years commonly attributed to Azithromycin in 30.95%, followed by Cloxacilllin in 26.19% in our study.

In another study from India, Staphylococcus aureus exhibited highest resistance for Tetracycline (92.4%), Co- trimoxazole (86.08%), Ampicillin (87.35%), and Gentamicin (56.97%). Higher occurrence of disease was noted among younger age groups, 46% among 0-10 years and 28% between 11-20 years.^1^

Azithromycin is commonly used in common cold, acne vulgaris even without prescriptions, which may have led to high resistance in our study, attributed to almost one third of cases. Cloxacillin on the other hand, is also commonly used over the counter. Larger multicenter studies or community studies may be needed to verify the actual status.

Limitation of this study was since the samples were taken from outpatients only; it may not reflect the real situation of community-acquired pyoderma in the community.

## CONCLUSIONS

Prevalence of community-acquired pyoderma is high among patients visiting dermatological outpatient departments of a tertiary care hospital compared to other studies. Antibiotic resistance of commonly used antibiotics are increasing and thus proper culture and sensitivity reports may be required to guide our treatment.

## Conflict of Interest

**None.**
